# Hepatobiliary long-term consequences of COVID-19: dramatically increased rate of secondary sclerosing cholangitis in critically ill COVID-19 patients

**DOI:** 10.1007/s12072-023-10521-0

**Published:** 2023-04-29

**Authors:** Silke Leonhardt, Christian Jürgensen, Josephine Frohme, Donata Grajecki, Andreas Adler, Michael Sigal, Julia Leonhardt, Julian M. Voll, Jan Matthias Kruse, Roland Körner, Kai-Uwe Eckardt, Hans-Joachim Janssen, Volker Gebhardt, Marc D. Schmittner, Pa-COVID-19 collaborative study group, Stefan Hippenstiel, Martin Witzenrath, Norbert Suttorp, Elisa T. Helbig, Lena J. Lippert, Paula Stubbemann, Pinkus Tober-Lau, David Hillus, Sascha S. Haenel, Alexandra Horn, Willi M. Koch, Nadine Olk, Mirja Mittermaier, Fridolin Steinbeis, Tilman Lingscheid, Bettina Temmesfeld-Wollbrück, Thomas Zoller, Holger Müller-Redetzky, Alexander Uhrig, Daniel Grund, Christoph Ruwwe-Glösenkamp, Miriam S. Stegemann, Katrin M. Heim, Ralf H. Hübner, Christian Drosten, Victor M. Corman, Bastian Opitz, Martin Möckel, Felix Balzer, Claudia Spies, Steffen Weber-Carstens, Chantip Dang-Heine, Michael Hummel, Georg Schwanitz, Uwe D. Behrens, Maria Rönnefarth, Sein Schmidt, Alexander Krannich, Saskia Zvorc, Jenny Kollek, Christof von Kalle, Jan Doehn, Christoph Tabeling, Linda Jürgens, Malte Kleinschmidt, Sophy Denker, Moritz Pfeiffer, Belén Millet Pascual-Leone, Luisa Mrziglod, Felix Machleidt, Sebastian Albus, Felix Bremer, Tim Andermann, Carmen Garcia, Philipp Knape, Philipp M. Krause, Liron Lechtenberg, Yaosi Li, Panagiotis Pergantis, Till Jacobi, Teresa Ritter, Berna Yedikat, Lennart Pfannkuch, Christian Zobel, Ute Kellermann, Susanne Fieberg, Laure Bosquillon de Jarcy, Anne Wetzel, Markus C. Brack, Moritz Müller-Plathe, Daniel Zickler, Andreas Edel, Britta Stier, Nils B. Müller, Philipp Enghard, Lucie Kretzler, Lil A. Meyer-Arndt, Linna Li, Isabelle Wirsching, Denise Treue, Dana Briesemeister, Jenny Schlesinger, Daniel Wendisch, Anna L. Hiller, Sophie Brumhard, Christian Frey, Hendrik Müller-Ide, Michael Bauer, Charlotte Thibeault, Florian Kurth, Leif Erik Sander, Tobias Müller, Frank Tacke

**Affiliations:** 1 Department of Hepatology and Gastroenterology, Charité-Universitätsmedizin Berlin, Corporate Member of Freie Universität Berlin, Humboldt-Universität zu Berlin, Campus Virchow Klinikum, Augustenburger Platz 1, 13353 Berlin, Germany; 2 Department of Hepatology and Gastroenterology, Charité-Universitätsmedizin Berlin, Corporate Member of Freie Universität Berlin, Humboldt-Universität zu Berlin, Campus Mitte, Charitéplatz 1, 10117 Berlin, Germany; 3 Department of Anesthesiology and Intensive Care Medicine, University Hospital Jena and Center for Sepsis Control and Care, University Hospital Jena, Am Klinikum 1, 07747 Jena, Germany; 4 Department of Nephrology and Medical Intensive Care, Charité-Universitäts-Medizin Berlin, Corporate Member of Freie Universität Berlin, Humboldt-Universität zu Berlin, Augustenburger Platz 1, 13353 Berlin, Germany; 5 Department of Anesthesiology, Intensive Care and Pain Medicine, BG Klinikum Unfallkrankenhaus Berlin gGmbH, Warener Strasse 7, 12683 Berlin, Germany; 6 Department of Anesthesiology and Intensive Care, Bundeswehrkrankenhaus, Berlin, Scharnhorststrasse 13, 10115 Berlin, Germany; 7 Department of Internal Medicine, Cardiology and Medical Intensive Care, Vivantes Hospital Spandau, Neue Bergstrasse 6, 13585 Berlin, Germany; 8 Department of Infectious Diseases and Respiratory Medicine, Charité-Universitätsmedizin Berlin, Corporate Member of Freie Universität Berlin, Humboldt-Universität zu Berlin, and Berlin Institute of Health, Campus Virchow Klinikum, Augustenburger Platz 1, 13353 Berlin, Germany; 9 German Centre for Lung Research (DZL), Gießen, Germany

**Keywords:** COVID-19 cholangiopathy, Secondary sclerosing cholangitis in critically ill patients, Ischemic cholangiopathy, COVID-19 long-term consequences, COVID-19 associated SSC-CIP

## Abstract

**Background:**

Increasing evidence suggests that secondary sclerosing cholangitis (SSC), which can lead to cirrhosis or liver failure, may be a hepatobiliary long-term complication of COVID-19. The aim of this study was to estimate the frequency and outcome of this COVID-19 sequela and to identify possible risk factors.

**Methods:**

This observational study, conducted at University Hospital Charité Berlin and Unfallkrankenhaus Berlin, Germany, involved hospitalized patients with COVID-19 pneumonia, including 1082 ventilated COVID-19 patients. We compared COVID-19 patients who developed SSC with a COVID-19 control group by univariate and multivariate analyses.

**Results:**

SSC occurrence after COVID-19 was observed exclusively in critically ill patients with invasive ventilation, albeit with extreme clustering among them. One in every 43 invasively ventilated COVID-19 patients developed this complication. Risk factors preceding the development of secondary sclerosing cholangitis in critically ill COVID-19 patients (SSC-CIP) were signs of systemic reduced blood oxygen supply (e.g., low PaO_2_/FiO_2_, ischemic organ infarctions), multi-organ failure (high SOFA score) at admission, high fibrinogen levels and intravenous ketamine use. Multivariate analysis confirmed fibrinogen and increased plasma lactate dehydrogenase as independent risk factors associated with cholangiopathy onset. The 1-year transplant-free survival rate of COVID-19-associated SSC-CIP was 40%.

**Conclusions:**

COVID-19 causes SSC-CIP in a substantial proportion of critically ill patients. SSC-CIP most likely develops due to severe tissue hypoxia and fibrinogen-associated circulatory disturbances. A significant increase of patients with SSC-CIP is to be expected in the post-COVID era.

**Supplementary Information:**

The online version contains supplementary material available at 10.1007/s12072-023-10521-0.

## Introduction

A novel coronavirus causing severe acute respiratory syndrome emerged in late 2019. This SARS-CoV-2 is a “classic” respiratory virus, which primarily affects the upper and lower respiratory system. More recent evidence has shown that coronavirus disease 2019 (COVID-19) is not limited to the airways, but also involves other organ systems, such as skin, kidneys, eyes, and endocrine organs as well as the cardiovascular and central nervous system [1–7]. Gastrointestinal symptoms are also common and liver involvement, in particular, is frequently observed. Liver function test (LFT) abnormalities are found in 14–65% of hospitalized patients with symptomatic COVID-19; they are more significant in severe cases and occur in up to 80% of fatal cases [8–10]. Early data from the first wave of the pandemic suggested that hepatocellular and mixed type patterns of liver involvement were the predominant form, whereas cholestatic patterns were less common [11–13].

However, as new clinical data emerge about COVID-19, there has been a growing focus on cholestatic complications of COVID-19, which are primarily associated with a severe course of COVID-19 [14]. Several experimental lines of evidence, including biliary organoid cultures, work with cell-lines, and analyses of the SARS-CoV-2 entry receptor ACE2 in human liver, indicate that SARS-CoV-2 can directly infect and replicate in liver cells, particularly cholangiocytes (i.e., biliary epithelial cells) [9]. From a clinical perspective, various authors have reported pronounced biliary tract destruction with the development of secondary sclerosing cholangitis in association with COVID-19 pneumonia, also termed “COVID-19 cholangiopathy” [15–20]. Previous experience with the occurrence of a secondary sclerosing cholangitis in conjunction with viral diseases was first gathered during the influenza A (H1N1) pandemic of 2009/2010 [21]. Based on these results, severe acute respiratory distress syndrome (ARDS) was established as an underlying condition for the development of secondary sclerosing cholangitis in critically ill patients (SSC-CIP).

To date (March 2023), more than 759 million confirmed cases of coronavirus disease 2019 have been reported worldwide (WHO Dashboard), and awareness of the long-term health effects of this disease is growing. Nevertheless, the cholestatic long-term consequences of COVID-19 are not well reflected in the current literature [22]. Acquired in the early course of intensive care, COVID-19 cholangiopathy persists from the acute phase of COVID-19 to the time of recovery. Patients with COVID-19 cholangiopathy remain seriously ill, even after complete pulmonary recovery.

The aim of this study was to determine whether COVID-19 promotes a cholangiopathy and if so, at what frequency. Under the hypothetical assumption that this cholangiopathy corresponds to SSC-CIP, we aimed to define risk factors for COVID-19-associated SSC-CIP by comparing these patients to a well-matched COVID-19 control group. To our knowledge, there is no such comparative analysis in the available literature.

## Methods

### Study design and cohort

This work is an ambidirectional observational study conducted and coordinated by the University Hospital Charité Berlin (“Charité”) in Berlin, Germany. The study is a sub-project embedded in the prospective observational PaCOVID-19 study, the characteristics of which have been described elsewhere [23]. As a tertiary university hospital, Charité provides care for hospitalized COVID-19 patients and, especially, critically ill COVID-19 patients. Briefly, we used a prospective design to estimate the frequency of SSC-CIP in patients with COVID-19 pneumonia. The sub-project’s study population consisted of adults (≥ 18 years) with COVID-19 pneumonia confirmed by PCR on nasopharyngeal swabs, who were admitted to Charité between March 1, 2020 and March 31, 2021, and who developed SSC-CIP. SSC-CIP is a highly symptomatic disorder of critically ill patients, who present with severe progressive, gamma-GT-enhanced cholestasis. As part of our hospital’s standardized treatment strategy for COVID-19 patients, cholestasis parameters were evaluated on admission and during follow-up of all of our COVID-19 patients, and every 1–2 days in ICU patients. Inclusion criteria for SSC-CIP were: A. COVID-19 patients with suspected SSC-CIP, defined as: A1. Increase in GGT > 3 times the upper limit of normal (ULN) and ALP > 1.5 × ULN early (4–9 days) after ICU admission (according to EASL definition "cholestasis"). A2. Progressive elevation of cholestasis parameters after A1. A3. Cholestasis peaks approximately 24 to 36 days after intubation. A4. Peak of GGT reaches > 15 × ULN and ALP > 5 × ULN (Table 1).

**Table 1:** Baseline and clinical characteristics of COVID-19 patients with (cases) and without SSC-CIP (controls)

	COVID-19 patients without SSC-CIP (control group)	COVID-19 patients with SSC-CIP	*p* value^1^
	*N* = 25	*N* = 25	
Male gender (%,n)	72% (18)	72% (18)	1.0
Age (years)			
Median [IQR]	62 [47.0–69.0]	59 [49.0–63.0]	0.466^**2**^
Mean ± SD	56.2 ± 17.9	56.6 ± 10.5	
BMI (kg/m^2^)			
Median [IQR]	28.1 [25.5–36.3]	29.4 (25.4–34.1]	0.544^**2**^
Obesity (BMI > 30 kg/m^2^)	44% (11)	48% (12)	1.0
ARDS	100%	100%	n.a
**Time interval, median [IQR]**			
Symptom onset to ICU admission (days)	7.0 [5.0–9.0]	6.0 [3.0–10.0]	0.402^**2**^
Symptom onset to intubation (days)	8.0 [6.0–10.0]	7.0 [4.0–11.0]	0.514^**2**^
**ABO/Rh blood group system**^†^			
Group A	57.1% (12/21)	36% (9/25)	0.159♯
Group B	4.8% (1/21)	24% (6/25)	0.159♯
Group AB	0% (0)	4% (1/25)	0.159♯
Group 0	38.1% (8/21)	36% (9/25)	0.159♯
Rh D	76.2% (16/21)	88% (22/25)	0.439♯
**Past history of**			
Liver disease	8% (2)	4% (1)	1.0
Arterial hypertension	52% (13)	76% (19)	0.140
Diabetes mellitus	24% (6)	44% (11)	0.232
Pulmonary disease	28% (7)	24% (6)	1.0
COPD	12% (3)	0% (0)	0.235
Asthma	4% (1)	8% (2)	1.0
OSAS	12% (3)	12% (3)	1.0
Sarcoidosis	0% (0)	4% (1)	1.0
Cardiac disease	36% (9)	20% (5)	0.462
Coronary artery disease	24% (6)	16% (4)	0.725
Others	12% (3)	4% (1)	0.609
Chronic kidney disease	8% (2)	12% (3)	1.0
Other chronic diseases	52% (13)	32% (8)	0.252
Pregnancy (during ICU stay)	4% (1)	4% (1)	1.0
**Parameters of cholestasis, median [IQR]**			
GGT, initial, U/L	**145.0 [88–178]**	**198.0 [170–281]**	**0.006**^**2**^
GGT, peak,U/L	238.0 [160–473]*^9^	2193.0 [1782–2522]*^25^	** < 0.001**^**2**^
ALP, initial, U/L,	**149.0 [108–168]**	**261.0 [201–311]**	** < 0.001**^**2**^
ALP, peak, U/L,	167.0 [132–201]*^6^	**1136.0 [903–1464**]*^30^	** < 0.001**^**2**^
Bilirubin, initial, mg/dL,	**0.6 [0.4–0.6]**	**3.0 [2.7–3.5]**	** < 0.001**^**2**^
Bilirubin, peak, mg/dL,	0.9 [0.7–1.2]*^4^	**14.0 [5.7–19.9**]*^35^	** < 0.001**^**2**^

*p*-values <0.05 were considered as significant and highlighted in bold

^†^No data from 4 control group patients

♯global testing of the entire group

^*^day of reaching the peak (after intubation)

*ALP* Alkaline phosphatase (normal range 40–130 U/L)*, ARDS* Acute respiratory distress syndrome, *Bilirubin* (normal range < 1.2 mg/dL), *BMI* Body mass index, *COPD* Chronic obstructive pulmonary disease, *GGT* Gamma-glutamyl transpeptidase (normal range 8–61/ U/L), *n.a.* not applicable, *OSAS* Obstructive sleep apnea syndrome*, SD* Standard deviation, *SSC-CIP* Secondary sclerosing cholangitis in critically ill patients, *ICU* Intensive-care unit, *IQR,* Interquartile range

^1^Fisher’s exact test unless otherwise stated

^2^ MW Mann–Whitney U test

If SSC-CIP was suspected (or uncertainly assignable cases, borderline cholestasis values, etc.), non-invasive differential diagnosis was performed (according to the recommendations of specialist societies, including imaging studies, such as ultrasound, CT, MRI, and MRCP), followed by ERC, if needed. B. The diagnosis of SSC-CIP was confirmed if imaging and/or autopsy revealed the following findings in addition to the aforementioned laboratory chemistry abnormalities: B1. Intrahepatic bile duct destruction (100% of cases), cholangiographic signs of secondary stenoses (in the area of necrotic wall destruction) with prestenotic dilatation (“beaded appearance”), and onset of bile duct injury in the liver periphery. B2. Biliary cast formation (76% of cases). Only confirmed cases were included in the study (23 × by ERC, 1 × by MRCP and 1 × by autopsy). Control subjects consisted of patients from the PaCOVID-19 study cohort, who were retrospectively sampled in a nested case–control fashion. The control group was carefully selected for age (± 5 years) and gender to produce a comparable age and gender distribution of the SSC-CIP and control group. The inclusion criteria for controls were as follows: confirmed SARS-CoV-2 pneumonia, age ≥ 18 years, invasive ventilation, no or only mild cholestasis initially (GGT < 3 × ULN and ALP < 1.5 × ULN), no progression to severe cholestasis (GGT peak of controls were median: 3.9 × ULN; and ALP peak median: 1.3 × ULN, see Table 1), improvement of mild cholestasis during ICU stay, no occurrence of cholestatic liver disease during follow-up (cut-off date: 30 April 2022; after the end of follow-up, the controls or their relatives were contacted by telephone and interviewed). Exclusion criteria for controls were: nasal mask or high-flow oxygen therapy, progressive cholestasis during ICU treatment, increase in GGT > 14 × ULN AP > 4.5 × ULN during the ICU stay (even if this was only detectable temporarily), new cholestatic liver disease in the follow-up period, and unclear bile duct changes in MRCP or ERC. All included patients (SSC-CIP and control group) met the criteria for the Berlin definition of ARDS [24].

As a tertiary university hospital and ARDS center, Charité provides the highest level of critical care for COVID-19 patients, including extracorporeal membrane oxygenation (ECMO) support (level 1 hospital). Accordingly, Charité treated some of the most severely ill COVID-19 patients, and many patients in our cohort were transferred to Charité from other hospitals after their condition worsened. To detect a resulting selection bias and, if any, to mitigate it, patients from a second treatment center (Unfallkrankenhaus Berlin) were also included in our analysis. The cases treated at this level 2 hospital (high level of critical care without ECMO) were recorded anonymously and retrospectively.

After the relevant study results were available, we subsequently included an additional control group (with comparable age and gender distribution as the other groups). To estimate the impact of typical COVID-19 pathophysiology on our results, we selected a control group of ARDS patients due to non-COVID-19 pneumonia. Their data were collected anonymously and retrospectively. Only targeted variables (PaO_2_/FiO_2_, fibrinogen) were recorded. These patients were invasively ventilated for viral or bacterial pneumonia and met the ARDS criteria (all of these controls had tested negative for COVID-19).

### Outcome measures

The primary outcome measure was the frequency of SSC-CIP in COVID-19 patients. For analysis of secondary outcome measures, we attempted to identify potential risk factors for SSC-CIP, especially those that might define this form of COVID-19 cholangiopathy as SSC-CIP.

### Data collection

Demographic and clinical data (e.g., comorbidities, medications, ventilation data, organ replacement therapy, and laboratory tests) of all patients were extracted from the patient records (for exact list, see supplementary material, Table S1). Any biologically plausible cause of bile duct damage was considered a potential risk factor for SSC-CIP in critically ill COVID-19 patients. Fifty-two potential risk factors were recorded (Supplementary table 1). Only those parameters that manifested before the onset of cholestasis were included in the analysis. Day 1 was defined as the day of intubation. According to the recommendations of the European Association for the Study of the Liver (EASL), the onset of cholestasis was defined as serum alkaline phosphatase (ALP) levels higher than 1.5 times the upper limit of normal (> 1.5 × ULN), gamma-glutamyl transpeptidase (GGT) levels > 3 × ULN, and bilirubin levels > 2 × ULN [25]. Lymphopenia was defined as a lymphocyte count of less than 1100 × 10^3^/mm^3^ based on other studies and local laboratory reference ranges [26]. Thrombocytopenia was defined as a platelet count of less than 150/nl.

### Statistical analysis

We compared the characteristics of COVID-19-related ARDS patients with and without SSC-CIP (control group). Continuous variables were expressed as mean ± standard deviation (SD) or median with interquartile range (IQR). The Mann–Whitney U test was used to examine the differences between the two groups. Categorical variables were expressed as percentages (%) and compared across the groups using Fisher's exact test. All, statistical tests were two-sided, and a p value < 0.05 were considered statistically significant. Kruskal–Wallis test was used to compare three different ARDS groups (Table 5). A multivariate analysis by means of logistic regression with stepwise selection (significance level *p* < 0.05) was performed to identify risk factors for the development of SSC-CIP. Considering the two relevant pathogenic hypotheses for SSC-CIP development (“toxic bile” and “biliary ischemia”), and to avoid overloading the model, only selected variables identified in the univariate analysis were included in the multivariate analysis. For survival analysis, we used the Kaplan–Meier method.

## Results

### Frequency of SSC-CIP among COVID-19 patients

In a large COVID-19 cohort (2849 patients with confirmed COVID-19 admitted to Charité), SSC-CIP occurred exclusively in invasively ventilated COVID-19 patients. Not a single case of secondary sclerosing cholangitis occurred in cases with mild COVID-19 or non-invasive ventilation. A total of 25 out of 1082 invasively ventilated COVID-19 patients were diagnosed with new-onset secondary sclerosing cholangitis (incidence: 2.3/100; 95%CI: 1.5–3.4), corresponding to a rate of one out of 43 mechanically ventilated COVID-19 patients. Data from two treatment centers were included in the analysis. The SSC-CIP incidence rate was 2.0 (95%CI: 1.2–3.1) at the level 1 center (the study site with admission of particularly seriously ill COVID-19 patients) and 4.2 (95%CI: 1.5–8.8) at the level 2 center. No significant difference in the incidence rate between the two centers could be detected, the incidence rate ratio (IRR, center 1 vs. center 2) was: 0.49 [0.19–1.49], *p* value: 0.146. Thus, there was no evidence of a selection bias due to the inclusion of more severely ill patients from the level 1 ARDS center.

### Baseline characteristics of COVID-19 patients with and without SSC-CIP (controls) were equivalent

To identify risk factors for the development of SSC-CIP, we included mechanically ventilated COVID-19 patients without evidence of SSC-CIP as the control group (comparable in age and gender distribution). COVID-19 patients with and without SSC-CIP had similar baseline characteristics (Table 1). Those with SSC-CIP were predominantly males (72%) with a median age of 59 years (range 26–71) and a predominance (48%) of obesity (BMI > 30 kg/m^2^). Comorbidities were common in the SSC-CIP group (88%) and the control group (80%). However, there were no significant differences between the groups in terms of the prevalence of hypertension, chronic lung disease, or other comorbidities.

### COVID-19 patients with SSC-CIP were more likely to receive ketamine

**Fig. 1:**
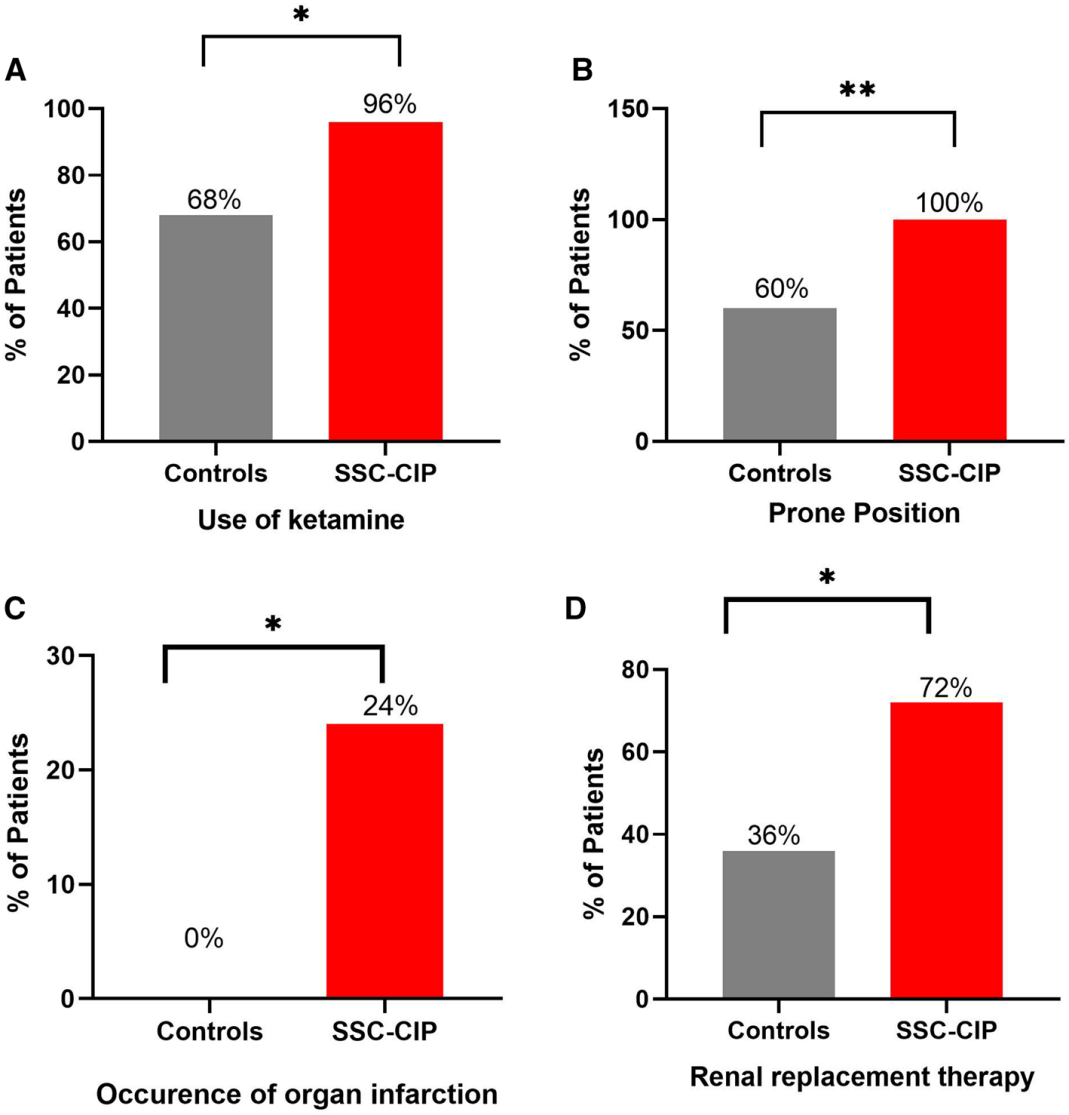
Selected risk factors for secondary sclerosing cholangitis in critically ill COVID-19 patients (prior the onset of cholestasis). Critically ill COVID-19 patients who developed SSC-CIP (*n* = 25, red bar graphs; SSC-CIP group) versus those who did not (*n* = 25; control group). Bar graphs show the proportion of patients with the selected risk factor in the group. **A** Ketamine use, **B** Prone position, **C** Organ infarction, and **D** Renal replacement therapy. * *p* < 0.05, ** *p* < 0.01

Associations between certain medications and cholangiopathies have been reported. Therefore, we compared the medications that the patients had been taking prior to hospital admission or in the ICU before onset of the first symptoms of SSC-CIP. The analysis revealed that the drugs taken by COVID-19 patients with and without SSC-CIP prior to hospital admission and in the initial ICU period were generally comparable (Table 2). However, ketamine use was significantly more common in the SSC-CIP group (*n* = 24; 96%) than in the control group (*n* = 17; 68%, *P* = 0.023, Fig. 1).

**Table 2:** Drug use prior to COVID-19 and medications during initial ICU course

	COVID-19 patients without SSC-CIP (control group)	COVID-19 patients with SSC-CIP	*p* value^1^
	*N* = 25	*N* = 25	
**Medications used prior to COVID-19: % (number) of patients**			
ARBs	41% (9/22)^‡^	31.8% (7/22)^‡^	0.755
Statins	22.7% (5/22)^‡^	31.8% (7/22)^‡^	0.736
Others	68.2% (15/22)^‡^	72.7% (16/22)^‡^	1.000
**Specific medications for COVID-19 prior to cholestasis: % (number) of patients**			
Corticosteroids	68% (17)	72% (18)	1.000
Hydrocortisone	24% (6)	12% (3)	0.232
Dexamethasone	32% (8)	60% (15)	0.088
Methylprednisolone	12% (3)	0% (0)	0.235
Antiviral agents/remdesivir	8% (2)	4% (1)	1.000
Additional immunosuppressants	0% (0)	20% (5)	0.050
Tocilizumab	0% (0)	12% (3)	0.235
Anakinra	0% (0)	8% (2)	0.490
Cytosorb	0% (0)	4% (1)	1.000
**Anticoagulants: % (number) of patients**			
Unfractionated heparin	52% (13)	52% (13)	1.000
Low molecular weight heparin	28% (7)	44% (11)	0.377
Direct thrombin inhibitors (Argatroban)	20% (5)	4% (1)	0.189
**Antibiotics: % (number) of patients**			
Piperacillin/tazobactam	64% (16)	72% (18)	0.762
Ampicillin/sulbactam	20% (5)	12% (3)	0.702
Fucloxacillin	0% (0)	4% (1)	1.000
Meropenem	32% (8)	20% (5)	0.520
Vancomycin	24% (6)	12% (3)	0.463
Cephalosporins	8% (2)	8% (2)	1.000
Macrolides	16% (4)	40% (10)	0.114
Ciprofloxacin	8% (2)	16% (4)	0.667
Linezolid	8% (2)	8% (2)	1.000
Cotrimoxazol	4% (1)	0% (0)	1.000
Others	4% (1)	4% (1)	1.000
**Anaesthetics: % (number) of patients**			
Ketamine	**68%** (**17**)	**96%** (**24**)	**0.023**
Esketamine alone	**36%** (**9**)	**48%** (**12**)	**0.041**
**Blood products: % (number) of patients**			
Red blood cell transfusion	36% (9)	28% (7)	0.762
Mean packed RBC units/ patient included above ± SD	2.6 ± 1.2	2.6 ± 1.8	0.783 ^2^
Fresh-frozen plasma	12% (3)	8% (2)	1.000
Mean FFP units/patient included above ± SD	9.0 ± 9.6	4.0 ± 0.0	0.554 ^2^

^‡^ Incomplete data

*ARBs* Angiotensin II receptor blockers,* RBC* Red blood cells, *FFP* Fresh frozen plasma, *SD *standard deviation, *SSC-CIP* Secondary sclerosing cholangitis in critically ill patients

^1^Fisher´s exact test if not stated else

^2^Mann-Whitney U test

### SSC-CIP development was associated with a significantly greater severity of hypoxemia and of COVID-19 disease

**Fig. 2:**
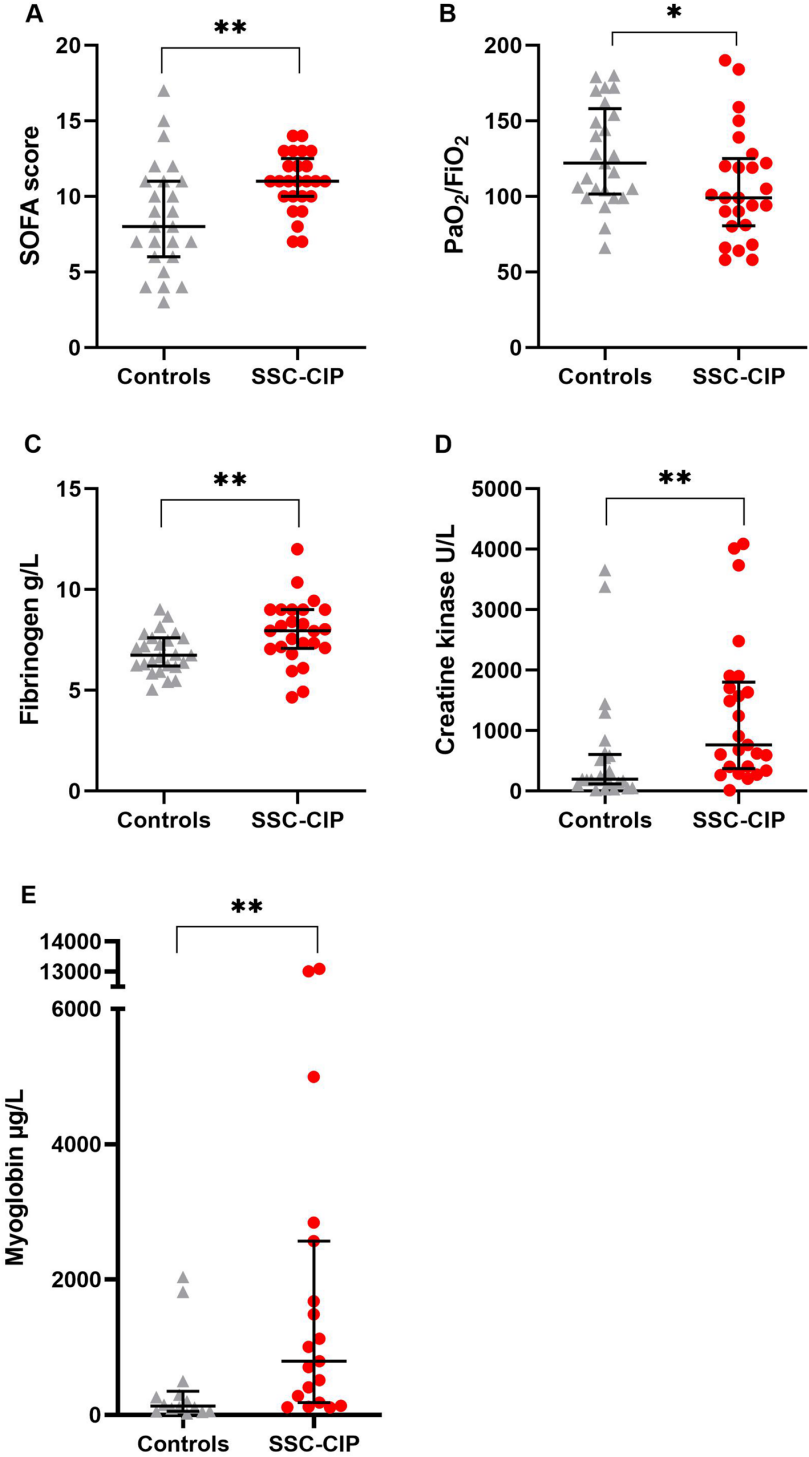
Risk factors for SSC-CIP on ICU admission. Critically ill COVID-19 patients who developed SSC-CIP (*n* = 25, red data points; *SSC-CIP group*) versus those who did not *(n* = 25, *control group).* Dots and triangles represent values for individual patients, horizontal lines indicate median values, and bars indicate the interquartile range. **A** SOFA (Sequential Organ Failure Assessment) score, **B** Oxygenation index (PaO_2_/FiO_2_), **C** Serum fibrinogen, **D** Serum creatine kinase, and **E** Serum myoglobin. * *p* < 0.05, ** *p* < 0.01

The SSC-CIP and control groups differed in terms of illness severity, as determined using Sequential Organ Failure Assessment (SOFA) score (Table 3, Fig. 2). The median SOFA score on ICU admission was 11.0 [IQR 10.0–12.0] in the SSC-CIP group vs. 8.0 [6.0–11.0] in the control group; the difference was statistically significant (*p* = 0.008). Accordingly, the COVID-19 patients who developed SSC-CIP had higher rates of acute renal failure (76% vs. 56%, n.s.) and renal replacement therapy (72% vs. 36%, *p* = 0.022). Although all patients in both groups had COVID-19-related ARDS, there was a significant difference in oxygenation index values between the two groups: the median PaO_2_/FiO_2_ on ICU admission was 99.0 mmHg [81.0–122.0] in patients who developed SSC-CIP vs. 122.0 mmHg [104.0–154.0] in those who did not (*p* = 0.034). Severe ARDS according to the Berlin definition occurred in thirteen SSC-CIP-positive vs. six SSC-CIP-negative cases. Hence, 13 SSC-CIP patients and 6 non-SSC-CIP controls required ECMO.

**Table 3:** Symptoms of organ failure, comparison of the two groups

	COVID-19 patients without SSC-CIP (control group)	COVID-19 patients with SSC-CIP	*p* value^1^
	*N* = 25	*N* = 25	
**Organ failure prior to cholestasis**			
SOFA score on admission			
Mean ± SD	**8.7 ± 3.6**	**10.9 ± 1.9**	**0.008**
Median [IQR]	**8.0** [**6.0–11.0**]	**11.0** [**10.0–12.0**]	
**Respiratory system**			
PaO_2_/FiO_2_ (mmHg)			
Mean ± SD	**127.3 ± 33.0**	**107.1 ± 36.4**	**0.034**^**2**^
Median [IQR]	**122.0 [104–154]**	**99.0 [81–122]**	
PEEP (cm H_2_O)			
Mean ± SD	14.5 ± 5.5	15.8 ± 3.0	0.435^**2**^
Median [IQR]	15.0 [12.0–17.0]	16.0 [14.0–18.0]	
ECMO % (*n*)	24% (6)	52% (13)	0.079
Prone Position % (*n*)	**60% (15)**	**100% (25)**	** < 0.001**
Severe ARDS % (*n*)	24% (6)	52% (13)	0.079
**Cardiovascular system**			
Use of vasopressors % (*n*) (norepinephrine)	100% (25)	100% (25)	n.a
Maximum dose of norepinephrine [µg/kg/min] Median [IQR]	0.1 [0.1–0.4]	0.2 [0.1–0.2]	0.285^**2**^
Episodes of MAP < 65 mmHg	76% (19)	69.6% (13/23)^§^	0.748
**Liver**			
Bilirubin median [IQR]	**0.6 [0.4–0.6]**	**3.0 [2.7–3.5]**	** < 0.001**^**2**^
**Coagulation**			
Platelets × 10^3^/μl median [IQR]	206 [155.0–297.0]	252.0 [221–312]	0.194^**2**^
VTE % (*n*)	20% (5)	24% (6)	1.000
PT median [IQR]	78.0 [66.0–95.0]	89.0 [69.0–98.0]	0.404^**2**^
**Kidneys**			
Acute kidney failure	52% (13)	76% (19)	0.140
Renal replacement therapy	**36% (9)**	**72% (18)**	**0.022**

*p*-values <0.05 were considered as significant and highlighted in bold

^§^Incomplete data from 2 patients

^¶^Including acute and acute-on-chronic kidney failure

*ARDS* Acute respiratory distress syndrome, *ECMO* Extracorporeal membrane oxygenation, *IQR* Interquartile range, *MAP* Mean arterial pressure, *n.a*. not applicable, *SD* Standard deviation, *SOFA score* Sequential organ failure assessment score, *SSC-CIP* Secondary sclerosing cholangitis in critically ill patients, *PEEP* Positive end-expiratory pressure, *PT* Prothrombin time, *VTE* Venous thromboembolism *PEEP* Positive end-expiratory pressure, *PT* Prothrombin time, *VTE* Venous thromboembolism

^1^Fisher's exact test unless otherwise stated

^2^Mann–Whitney *U* test

### Cytokine storm and coagulation parameters were roughly comparable between the groups

We also sought evidence of differences in classical inflammation parameters between the two groups that would support the toxic bile concept (Table 4). Mechanically ventilated COVID-19 patients with and without SSC-CIP had comparable initial inflammation levels on admission (Table 4), as indicated by C-reactive protein (CRP), procalcitonin (PCT), interleukin-6 (IL-6), and ferritin. Clinical and laboratory parameters indicative of a hypercoagulable state include D-dimers, fibrinogen, prothrombin time (PT), platelet count, and the rate of thrombotic events. Median plasma fibrinogen levels on admission were significantly higher in the patients who developed SSC-CIP than in those who did not (8.0 g/L [IQR 7.1–9.0] vs. 6.7 g/L [IQR 6.2–7.6]; *p* = 0.008). However, the other coagulation parameters mentioned above did not differ significantly between the groups.

**Table 4:** Selected risk factors for SSC-CIP, results of univariate analysis

	COVID-19 patients without SSC-CIP (control group)	COVID-19 patients with SSC-CIP		*p* value^1^
	*N* = 25	*N* = 25	OR (95%-CI)	
**Cytokine storm (related to toxic bile concept)**				
IL-6, initial, ng/L	161.0 [69.3–371.0]*	145.0 [56.9–421.0]*	1.001 (0.999–1.002)	0.690
IL-6, peak, ng/L	228.0 [64.0–689.0]*	502.0 [193.0–1180.0]*	1.0000 (0.9999–1.00006)	0.092
CrP, initial, mg/L	170.0 [107.0–256.0]*	220.0 [118.6–295.3]*	1.002 (0.997–1.007)	0.574
CrP, peak, mg/L	339.0 [267.0–381.0]*	349.0 [277.9–415.5]*	1.002 (0.997–1.007)	0.347
PCT, initial, µg/L	0.7 [0.2–1.4]*	0.8 [0.1–1.2]*	0.928 (0.802–1.073)	0.946
PCT, peak, µg/L	3.0 [0.8–8.6]*	5.8 [2.9–7.5]*	1.004 (0.976–1.033)	0.135
Lymphocytes/nL, initial	0.9 [0.6–1.2]*	0.6 [0.4–1.3]*	0.629 (0.328–1.204)	0.159
Lymphocytes/nL, Nadir	0.7 [0.4–1.1]*	0.5 [0.3–0.7]*	**0.491 (0.215–1.121)**	**0.030**
Lymphocytopenia, % (n)	72% (18)	92% (23)		0.138^2^
Ferritin, initial, µg/L	1055.0 [675.0–1460]*	1431.5 [841.5–2050.5]*	1.0001 (0.9997–1.000)	0.159
Ferritin peak, µg/L	**1508.6** [**846.0–2992**]*	**5709.5** [**3044.0–10,408.5**]*	**1.0001 (1.0001–1.003)**	** < 0.001**
**Factors associated with tissue hypoxia/ischemia**				
Organ infarction, % (*n*)	**0% (0)**	**24% (6)**		**0.022**^**2**^
PaO_2_/FiO_2_ (mmHg)	122.0 [104.0–154.0]*	99.0 [81.0–122.0]*	**0.984 (0.969–1.000)**	**0.034**
CK, initial, U/L	199.0 [134.0–585.0]*	765.0 [403.0–1706.0]*	**1.0005 (1.00004–1.001)**	**0.001**
CK, peak, U/L	585 [248.0–1395.0]*	1488 [749.0–2496.0]*	**1.0004 (1.00005–1.0008)**	**0.004**
LDH, initial, U/L	456.0 [345.0–619.0]*	543.0 [490.0–655.0]*	1.003 (1.000–1.007)	0.055
LDH, peak, U/L	619.0 [450.0–759.0]*	676.0 [592.0–770.0]*	1.000 (0.999–1.000)	0.184
Myoglobin, µg/L	133.5 [57.0–300.0]*^†^	795 [183.8–2570.0]*^‡^	**1.0002 (0.99998–1.0004)**	**0.006**
Fibrinogen, initial, g/L	6.7 [6.2–7.6]*	8.0 [7.1–9.0]*	**1.671 (1.133–2.462)**	**0.008**
**Hypercoagulable state**				
D-dimer, initial, mg/L	2.4 [1.4–4.4]*	1.9 [1.1–4.5]*	0.967 (0.869–1.077	0.503
D-dimer, peak, mg/L	10.7 [3.7–20.0]*	11.6 [5.9–20.7]*	1.015 (0.960–1.073)	0.638
min. Platelets × 10^3^/μl	156.0 [120.0–187.0]*	130.0 [75.0–183.0]*	0.998 (0.992–1.005)	0.332
Thrombocytopenia: % (n)	44% (11)	60% (15)		0.396^2^
PT	78.0 [66.0–95.0]*	89.0 [69.0–98.0]*	1.013 (0.989–1.037)	0.404
Fibrinogen, initial, g/L	**6.7** [**6.2–7.6**]*	**8.0** [**7.1–9.0**]*	**1.671 (1.133–2.462)**	**0.008**

*p*-values <0.05 were considered as significant and highlighted in bold

*CI* Confidence Interval, *CK* Creatine kinase, *CRP* C-reactive protein*, IL-6* Interleukin-6, *IQR* Interquartile range*, LDH* Lactate dehydrogenase*, OR* Odds ratio*, SOFA score* Sequential organ failure assessment score, *SSC-CIP* Secondary sclerosing cholangitis in critically ill patients, *PT* Prothrombin time, *PCT* Procalcitonin

^1^Mann–Whitney *U* test unless otherwise stated

^2^Fisher´s exact test

^*^median [IQR]

^†^No data from 11 patients

^‡^No data from 6 patients

### Diminished blood and oxygen supply to the tissues precedes the development of SSC-CIP

Organ infarctions are considered a consequence of diminished blood or oxygen supply to the tissues. Interestingly, the number of organ infarctions (heart, testis, spleen, and intestinal ischemia) was significantly higher in the SSC group (24% vs. 0%, *p* = 0.022). We noted striking differences in hypoxia markers between the groups. For example, initial hypoxia, as measured using the oxygenation index (see above) was significantly more severe in SSC-CIP patients than in controls. Moreover, COVID-19 patients who developed SSC-CIP had significantly higher creatine kinase (CK) levels than the controls. CK elevation occurred in 96% of SSC-CIP-positive patients compared to 60% of SSC-CIP-negative controls. Likewise, median myoglobin values were significantly higher in SSC-CIP patients, and there was a trend toward higher LDH values at ICU admission (*p* = 0.055) in the SSC-CIP group. Fibrinogen is not only a parameter of coagulation; its effect goes beyond that. High fibrinogen levels are associated with increased plasma viscosity, which can impair organ perfusion, leading to ischemia and infarction. Our COVID-19 patients who developed SSC had significantly higher fibrinogen levels (Table 4).

### Fibrinogen and LDH are independent risk factors for SSC-CIP development

Multivariable logistic regression analysis was performed to identify independent risk factors for the development of SSC-CIP in ventilated COVID-19 patients. The analysis revealed that high levels of fibrinogen (odds ratio [OR] 2.05, 95% confidence interval [CI]: 1.255 to 3.344, *p* = 0.004) and LDH (OR 1.005, 95% CI: 1.0004 to 1.009, *p* = 0.033) were independent risk factors for the development of SSC-CIP (Table S2). No independent associations were found for oxygenation index (PaO_2_/FiO_2_), SOFA score, or CK. Also, no significant associations were observed for inflammation markers like CRP, PCT, ferritin, IL-6, and lymphopenia.

### Comparison with a non-COVID-19 pneumonia control group

COVID-19 patients in the two subgroups with and without SSC-CIP had significantly higher median fibrinogen levels (8.0 g/L [IQR 7.1–9.0], and 6.7 g/L [IQR 6.2–7.6], respectively) than patients with non-COVID-19-related ARDS (4.5 g/L [IQR 2.7–5.2], *p* < 0.001) (Table 5). Likewise, hypoxemia was more pronounced in COVID-19 patients who developed SSC-CIP than in patients with non-COVID-19 ARDS (107.1 ± 36.4 vs. 134.3 ± 62.9). The difference was not significant (*p* = 0.077), but this is likely due to targeted selection of the control group (ARDS) and group size.

**Table 5:** Selected variables of different ARDS patients: Non-COVID-19 ARDS vs. COVID-19 ARDS w/o SSC-CIP vs. COVID-19 ARDS with SSC-CIP

	Non-COVID-19 associated ARDS *N* = 50	COVID-19 associated ARDS		*P* value
		Controls *N* = 25	SSC-CIP *N* = 25	
**Gender**				
Male	36	18	18	1.00
Female	14	7	7	
**Age**				
Mean ± SD	55.9 ± 15.0	56.2 ± 17.9	56.6 ± 10.5	
Median [IQR]	60.0 [45.0–67.0]	62.0 [47.0–69.0]	59.0 [49.0–63.0]	0.798
Fibrinogen, initial, g/L, median	**4.5 [2.7–5.2]**	**6.7 [6.2–7.6]**	**–**	** < 0.001**
		**–**	**8.0 [7.1–9.0]**	** < 0.001**
		**7.3 [6.3–8.2]**		** < 0.001**
PaO_2_/FiO_2_ (mmHg) Mean ± SD	134.3 ± 62.9	127.3 ± 33.0	-	0.657
		-	107.1 ± 36.4	0.077
		117.2 ± 35.9		0.416

*p*-values <0.05 were considered as significant and highlighted in bold

*ARDS* Acute respiratory distress syndrome, *IQR* Interquartile range, *SD* Standard deviation, *SSC-CIP* Secondary sclerosing cholangitis in critically ill patients

### Outcome of SSC-CIP patients

**Fig. 3:**
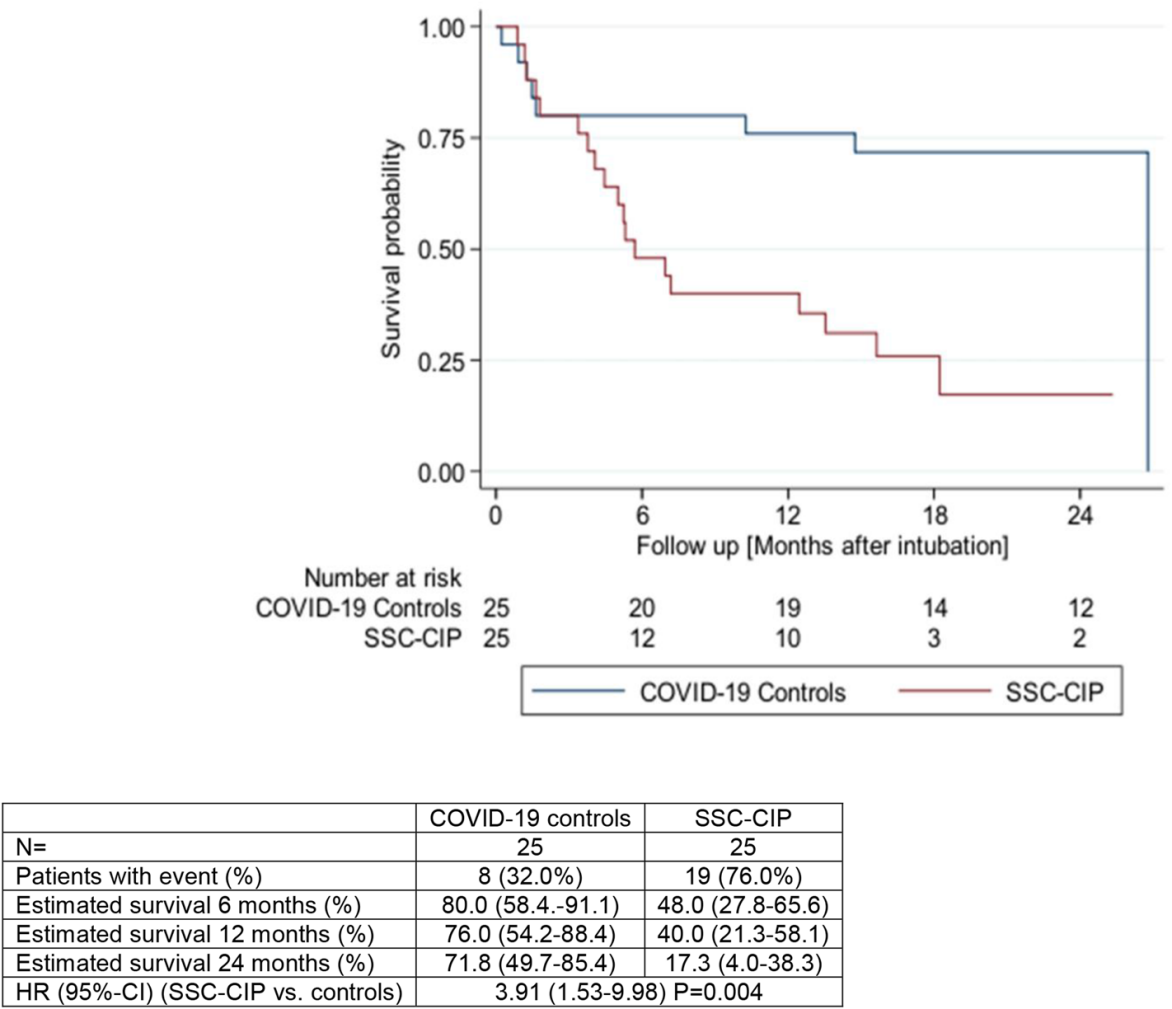
Kaplan–Meier survival analysis of COVID-19 patients with and without SSC-CIP. Critically ill COVID-19 patients who developed SSC-CIP (*n* = 25 red; SSC-CIP) versus those who did not (*n* = 25, blue; COVID-19 controls)

The length of ICU stay differed significantly between the SSC-CIP group and the control group (median: 65.0 [44.0–94.0] days vs. 28.0 [19.0–43.0] days, *p* < 0.001). COVID-19 patients who developed SSC-CIP had a numerically greater risk of death in the ICU (36% vs. 16%, n.s.). The survival of the SSC-CIP group was significantly worse than that of the COVID-19 control group without SSC-CIP [HR 3.91 (1.53–9.98) *p* = 0.004], as reflected by a median survival time of 5.7 months vs. 26.8 months and an estimated survival rate of 40.0% (21.3–58.1) vs. 76.0% (54.2–88.4), respectively, after 1 year (Fig. 3).

## Discussion

### Frequency and outcome

The data from this study draw attention to a clinically relevant hepatobiliary long-term consequence of COVID-19: COVID-19 increases the risk of secondary sclerosing cholangitis (SSC). SSC development was observed exclusively in invasively ventilated, critically ill COVID-19 patients (SSC-CIP). This cohort study revealed a dramatically increased rate of SSC-CIP in these patients and suggests that this complication is more likely due to established risk factors for SSC-CIP rather than to viral tropism or to a specific SARS-CoV-2-related etiology. Our observations confirm and extend the findings of recent case reports [15–18]. We found that SSC-CIP occurs at a rate of one in every 43 ICU patients on invasive ventilation. Compared to recently published data from the pre-pandemic era (1 case of SSC per 1990 ventilated patients, incidence: 0.05/100; 95%CI: 0.001–0.280) [27], the incidence of SSC-CIP is more than 46 times higher in critically ill COVID-19 patients (IRR 46.0; 95%CI: 7.5–1887.8). This is of great relevance in view of the resulting burden on healthcare systems around the world after the COVID-19 pandemic. The long and often complicated course of the disease and limited therapeutic options pose major challenges to hepatologists. Liver transplantation is the only curative treatment option, and previous reports indicate that up to 22% of all SSC-CIP patients require transplantation [15–18, 28]. Thus, an increasing need for liver transplantation in these relatively young post-COVID-19-SSC patients (mean age 56 years) can be expected, posing new challenges for transplantation medicine. Without transplantation, patients with COVID-19-associated SSC-CIP have a poor prognosis: only 40.0% of our patients were alive 1 year after SSC-CIP onset.

The clinical features of COVID-19-related SSC-CIP closely resemble those of our previous non-COVID cohort [29]. The available clinical data provide little-to-no evidence that post-COVID-19 SSC is a new disease entity originating primarily in the bile ducts, e.g., by direct SARS-CoV-2-mediated damage to cholangiocytes [16]. Instead, the disease shows most clinical and endoscopic features of classical secondary sclerosing cholangitis in critically ill patients (supplementary Fig. 1).

### Causes of SSC-CIP in COVID-19 patients

The extreme clustering of SSC-CIP among COVID-19 patients with ARDS raises the question of why these patients have a particular predisposition to SSC-CIP. Currently, there are two main concepts that might explain the pathophysiological mechanisms leading to SSC-CIP: The first theory is that hyperinflammation and a cytokine storm during critical illness may induce a dysregulation of the hepatobiliary transporter system, resulting in an altered bile composition (toxic bile concept). The second is that local ischemia of the biliary epithelium occurs during critical illness, leading to subsequent destruction of the bile ducts (ischemia concept) [27].

#### The “toxic bile concept” does not explain the increased incidence of SSC in critically ill COVID-19 patients

Inflammation biomarker levels in the present study were higher than those for critically ill COVID-19 patients in other studies [30]. This applies to our control group as well as to the SSC-CIP group. SARS-COV-2 triggers a cytokine storm, characterized by excessive elevation of interleukin-1β, interleukin-6, IP-10, TNF, interferon-γ, macrophage inflammatory protein (MIP) 1α and 1β, and vascular endothelial growth factor (VEGF). In COVID-19, IL-6 is the most prominently elevated cytokine and elevated IL-6 levels are strongly associated with poorer outcomes [31, 32]. Cytokine storm-induced dysregulation of the hepatobiliary transporter system can lead to impaired bile flow at the level of the small bile ducts (ductular cholestasis) or impaired uptake, transport and excretion of bile acids and bilirubin by the hepatocytes (hepatocellular cholestasis) [33–36]. Theoretically, the altered bile composition due to excessive cytokine release might jeopardize the integrity of bile duct epithelia. However, we found no evidence of significant differences in laboratory markers of cytokine storm (e.g., plasma levels of IL-6, CRP, PCT, and initial ferritin) between ventilated COVID-19 patients with and without SSC-CIP. This is in agreement with the other publications [14]. Thus, our results do not support the theory of cytokine-mediated bile duct destruction in SSC-CIP development in COVID-19 patients.

#### The “ischemia concept” can explain the increased incidence of SSC in critically ill COVID-19 patients

Shock and systemic hypoxia as well as local circulatory disturbances in the supplying peribiliary plexus (PBP) can contribute to bile duct ischemia in critically ill patients. Vasculitis, microthrombi, and hyperviscosity can lead to impaired blood flow in the PBP. Our COVID-19 patients who developed SSC-CIP had significantly more severe initial hypoxia, as measured using PaO_2_/FiO_2_ as a surrogate marker. As a consequence of severe hypoxia in COVID-19-related ARDS, the oxygen supply to the tissues is insufficient. Global tissue hypoxia may promote the development of multi-organ failure (MOF). As expected, SOFA scores on ICU admission, a measure of multi-organ failure, were significantly higher in SSC-CIP patients than in controls. The need for organ support as an expression of MOF differed accordingly. Renal replacement therapy due to acute kidney injury in the initial ICU course was significantly more common in COVID-19 patients with SSC-CIP than in those without it. To obtain further evidence of general tissue hypoxia, we compared the frequency of organ infarctions between the two groups. COVID-19 patients with SSC-CIP had significantly more organ infarctions (e.g., intestinal ischemia, splenic and testicular infarction) than the controls. Taken together, our results suggest that bile duct ischemia in the context of initial hypoxemic multi-organ failure may be a trigger mechanism precipitating SSC-CIP in COVID-19 patients. The multivariate analysis did not corroborate the results of the univariate analysis regarding the association of disease severity (PaO_2_/FiO_2_ and SOFA score) with the occurrence of SSC-CIP. The large number of variables in combination with the small sample size may hamper the power of the multivariate analysis for this question.

The results of our univariate and multivariate analyses indicate that fibrinogen plays a special role in the development of SSC-CIP. “Hyperfibrinogenemia” is associated with hyperviscosity, especially in COVID-19 patients [37]. Increased susceptibility of the bile ducts to hyperviscosity is a well-known phenomenon in the context of liver transplantation. Hyperviscosity is associated with ischemic bile duct lesions [38] as it causes disturbances of the microvascular circulation and tissue hypo-perfusion, damages the endothelium, and poses a risk factor for thrombosis. Evidence regarding the role of the vascular component in the pathology of COVID-19 is compelling [39, 40]. The blood of critically ill COVID-19 patients often has a unique stickiness, and the characteristics of COVID-19-associated coagulopathy are distinctly different from those ever seen in other critical illnesses [41, 42]. Subsequent thrombotic events like thrombi in the microvasculature are a hallmark of COVID-19. Tsutsumi et al. observed an association between elevated fibrinogen levels and liver involvement in COVID-19 and concluded that liver dysfunction in COVID-19 patients might be induced by microvascular thrombosis [43]. This hypothesis is supported by the results of a histopathological study by Kondo et al. [44]. Although the exact mechanism has yet to be proven, hyperfibrinogenemia may contribute to hypoxemia of the bile ducts and is an independent risk factor for SSC-CIP development in critically ill patients.

### Other possible mechanisms

Our COVID-19 patients who developed SSC-CIP received ketamine significantly more often before the onset of cholestasis (96%) than those in the control group (68%). These data are in line with our previous observations in non-SSC-CIP COVID-19 patients [27]. A possible association between ketamine use and cholangiopathies in COVID-19 patients was also reported in a multicenter study by French researchers [18,45]. The fact that ketamine can cause bile duct damage is known from numerous publications, especially from Asian countries, but the underlying mechanisms are unclear. Based on the results of our study, an unfavorable effect of ketamine on the development of SSC-CIP cannot be excluded. However, as one patient in our SSC-CIP group did not receive ketamine, it seems unlikely that ketamine was the sole cause of SSC-CIP development. It is also possible that the correlation is due to confounding factors, because COVID-19 patients with severe refractory ARDS tend to require deeper sedation, often in combination with ketamine, to enable prone positioning for escalation of treatment. However, the findings suggesting an association between ketamine and SSC-CIP need to be validated in future studies. Similarly, prone positioning was used significantly more often in patients with SSC. Prone positioning may further worsen the blood and oxygen supply to the bile ducts. On the other hand, it is notable that especially those patients with severe hypoxia who respond inadequately to conservative ventilation strategies are ventilated in prone position. Therefore, the presence of confounding is conceivable. These questions might be better addressed in studies involving patients who develop SSC-CIP as a result of prolonged shock rather than hypoxia.

According to our data, positive end-expiratory pressure (PEEP) and catecholamines do not appear to be independent risk factors for the development of SSC-CIP. Likewise, our results regarding the use of various medications do not support the hypothesis of drug-related cytotoxic effects on biliary epithelia. As the first controlled trial of SSC-CIP, our study provides important insights into the general pathogenetic mechanisms of SSC-CIP and evidence in support of the ischemic bile duct damage hypothesis favored by most authors.

### Others

The fact that COVID-19-associated SSC-CIP has not been previously reported in Asia may suggest a genetic predisposition. However, the fact that our SSC-CIP cohort included one patient of Asian origin and one of African origin does not support this assumption.

## Conclusion

Critically ill COVID-19 patients have an increased risk of developing secondary sclerosing cholangitis (SSC-CIP). This hepatobiliary long-term consequence of COVID-19 is common, affecting one in every 43 invasively ventilated patients. Our comparison of COVID-19 patients with non-COVID-19 ARDS controls suggests that the typical COVID-19 milieu of fibrinogen elevation in conjunction severe hypoxia may be conducive to the development of an SSC-CIP. This would also explain the jump in incidence of SSC-CIP due to COVID-19. Our study indicates that post-COVID-19 SSC-CIP is a hypoxic/ischemic event that may possibly be exacerbated by fibrinogen-associated hyperviscosity. In view of its high incidence and poor survival rate, COVID-19-associated SSC-CIP poses a major challenge for hepatologists.

### Supplementary Information

Below is the link to the electronic supplementary material.

Supplementary table S1: Potential risk factors for COVID-19-associated SSC-CIP identified in the study. Supplementary file1 (DOCX 97 KB)

Supplementary table S2: Results of multivariate logistic regression analysis. Supplementary file2 (DOCX 49 KB)

Supplementary figure S1: Typical cholangiographic findings in COVID-19 patients with SSC-CIP. Note the destruction and disappearance of the intrahepatic bile ducts. Intrahepatic segmental bile ducts (right lobe>> left liver lobe) show contour irregularities and interruptions. Supplementary file3 (JPG 417 KB)

Supplementary material: List of collected data. Supplementary file4 (DOCX 122 KB)

## Data Availability

The datasets generated and/or analyzed during the current study are available from the corresponding author on reasonable request.

## Abbreviations

ACE: Angiotensin-converting enzyme
ALP: Alkaline phosphatase
ARB: Angiotensin-receptor blocker
ARDS: Acute respiratory distress syndrome
BMI: Body mass index
CI: Confidence interval
CK: Creatine kinase
COPD: Chronic obstructive pulmonary disease
COVID-19: Coronavirus disease 2019
CRP: C-reactive protein
D-dimer: Dimerized plasma fragment D
EASL: European Association for the Study of the Liver
ECMO: Extracorporeal membrane oxygenation
ERC: Endoscopic retrograde cholangiography
FFP: Fresh-frozen plasma
GGT: Gamma-glutamyl transpeptidase
ICU: Intensive-care unit
IL-1, -6 or 10: Interleukin-1, -6 or 10
IP-10: Serum IFN-γ-induced protein 10
IQR: Interquartile range
IRR: Incidence rate ratio
LDH: Lactate dehydrogenase
LFT: Liver function test
MAP: Mean arterial pressure
MIP: Macrophage inflammatory protein
MOF: Multiorgan failure
MRCP: Magnetic resonance cholangiopancreatography
OSAS: Obstructive sleep apnea syndrome
PBP: Peribiliary plexus
PCR: Polymerase chain reaction
PCT: Procalcitonin
PEEP: Positive end-expiratory pressure
PT: Prothrombin time
RBC: Red blood cells
SD: Standard deviation
SOFA score: Sequential organ failure assessment score
SSC-CIP: Secondary sclerosing cholangitis in critically ill patients
TNF α: Tumor necrosis factor α
U/L: Units per liter
ULN: Upper limit of normal
VEGF: Vascular endothelial growth factor
VTE: Venous thromboembolism
