# Supplementary material for: Hepatobiliary long-term consequences of COVID-19: dramatically increased rate of secondary sclerosing cholangitis in critically ill COVID-19 patients
Source: Hepatol Int. 2023 Apr 29;17(6):1610–25. doi: 10.1007/s12072-023-10521-0 (PMC10148013; doi:10.1007/s12072-023-10521-0)
Supplement: Supplementary file 1 — Supplementary table S1: Potential risk factors for COVID-19-associated SSC-CIP identified in the study. Supplementary file1 (DOCX 97 KB) [file 12072_2023_10521_MOESM1_ESM.docx]

| 1 | BMI (kg/m²) |
| --- | --- |
| 2 | Liver disease |
| 3 | Arterial hypertension |
| 4 | Diabetes mellitus |
| *5* | Pulmonary disease |
| 6 | Cardiac disease |
| 7 | Chronic kidney disease |
| 8 | Other chronic diseases |
| 9 | Pregnancy |
|  | ***Medications used prior to COVID-19*** |
| 10 | ARBs |
| 11 | Statins |
| 12 | Others |
| 13 | ABO blood group system |
| 14 | Rh blood group system |
|  | ***Specific medications for COVID-19 prior to cholestasis*** |
| 15 | Corticosteroids |
| 16 | Antiviral agents/remdesivir |
| 17 | Cytosorb |
| 18 | Hydrochloroquin |
| 19 | Anakinra |
| 20 | Tocilizumab |
| 21 | Anticoagulants (prior to cholestasis) |
| 22 | Antibiotics |
| 23 | Anaesthetics (Ketamine prior to cholestasis) |
| 24 | **Blood products**: Red blood cell transfusion |
| 25 | **Blood products:** Fresh frozen plasma |
| 26 | Need for vasopressors |
| 27 | SOFA score |
| 28 | PaO_2_/FiO_2_ |
| 29 | PEEP |
| 30 | ARDS |
| 31 | Prone Position |
| 33 | ECMO |
| 34 | Acute kidney failure |
| 35 | Renal replacement therapy |
| 36 | Episodes of MAP<65 mmHg |
| 37 | Thrombotic events |
| 38 | Organ infarction |
| 39 | IL-6, ng/L |
| 40 | D-dimer, mg/L |
| 41 | Platelets×10^3^/μl |
| 42 | Platelets, Nadir |
| 43 | Prothrombin time |
| 44 | Fibrinogen, g/L |
| 45 | CrP, mg/L |
| 46 | PCT, µg/L |
| 47 | Lymphocytes/nL |
| 48 | Lymphocytes/nL, Nadir |
| 49 | Ferritin, µg/L |
| 50 | CK, U/L |
| 51 | LDH, U/L |
| 52 | Myoglobin, µg/L |
