# Supplementary material for: Hepatobiliary long-term consequences of COVID-19: dramatically increased rate of secondary sclerosing cholangitis in critically ill COVID-19 patients
Source: Hepatol Int. 2023 Apr 29;17(6):1610–25. doi: 10.1007/s12072-023-10521-0 (PMC10148013; doi:10.1007/s12072-023-10521-0)
Supplement: Supplementary file 2 — Supplementary table S2: Results of multivariate logistic regression analysis. Supplementary file2 (DOCX 49 KB) [file 12072_2023_10521_MOESM2_ESM.docx]

| **Table S 2 Results of multivariate logistic regression (stepwise selection)** | | | | | |
| --- | --- | --- | --- | --- | --- |
|  | **OR** | | **95%-CI** | | **P**  **value** |
|  |  | |  | |  |
| **Selected variables (p<0.05):** | |  | |  |  |
| Fibrinogen, initial, g/L | | **2.049** | | **1.255; 3.344** | **0.004** |
| LDH, initial, U/L | | **1.005** | | **1.0004; 1.009** | **0.033** |

*LDH* Lactate dehydrogenase*, CI* confidence interval*, OR* Odds ratio
