# Supplementary figures and images for: Hepatobiliary long-term consequences of COVID-19: dramatically increased rate of secondary sclerosing cholangitis in critically ill COVID-19 patients
Source: Hepatol Int. 2023 Apr 29;17(6):1610–25. doi: 10.1007/s12072-023-10521-0 (PMC10148013; doi:10.1007/s12072-023-10521-0)

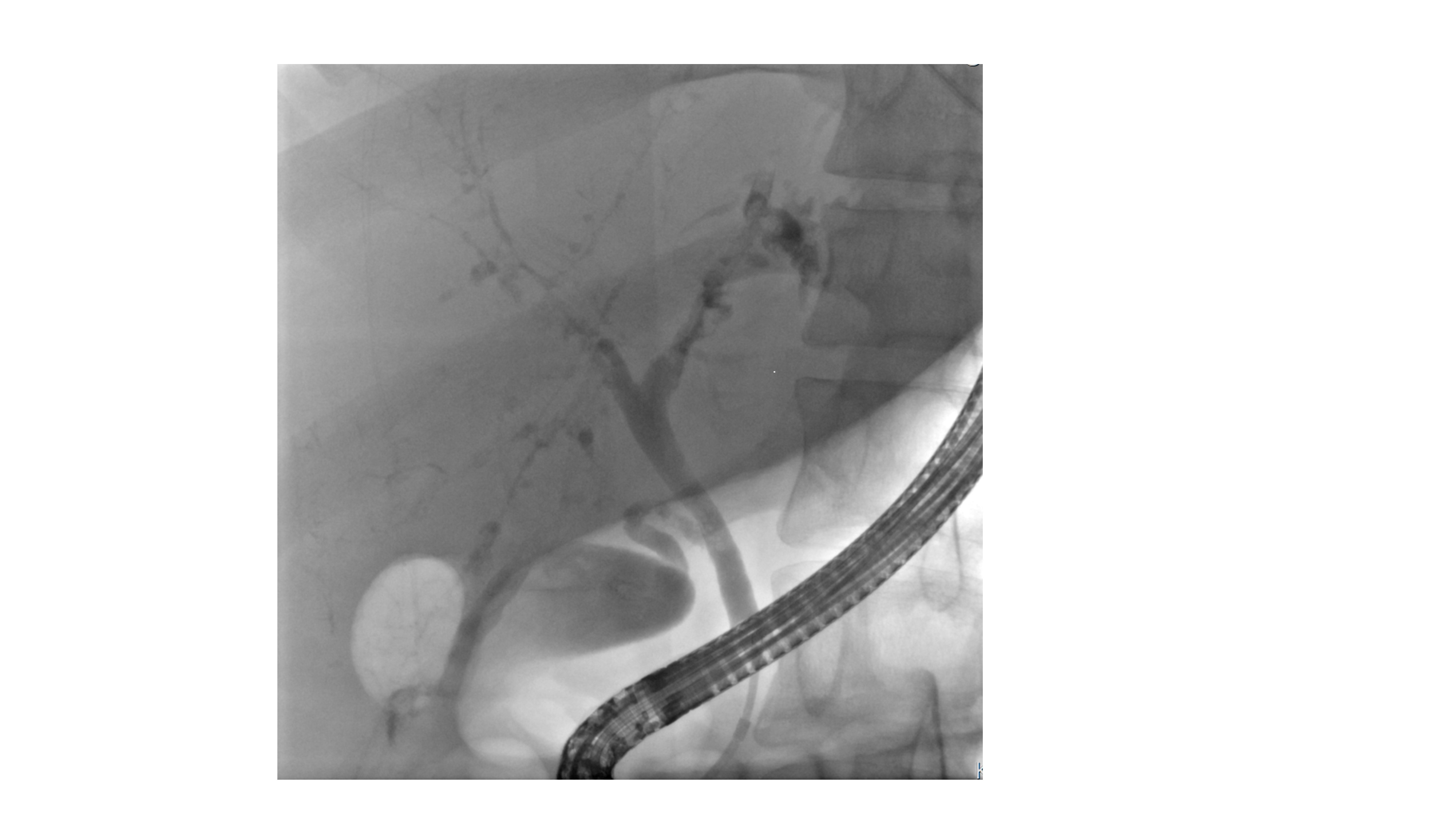

Supplement: Supplementary file 3 — Supplementary figure S1: Typical cholangiographic findings in COVID-19 patients with SSC-CIP. Note the destruction and disappearance of the intrahepatic bile ducts. Intrahepatic segmental bile ducts (right lobe>> left liver lobe) show contour irregularities and interruptions. Supplementary file3 (JPG 417 KB) [file 12072_2023_10521_MOESM3_ESM.jpg]
