# Supplementary material for: Hepatobiliary long-term consequences of COVID-19: dramatically increased rate of secondary sclerosing cholangitis in critically ill COVID-19 patients
Source: Hepatol Int. 2023 Apr 29;17(6):1610–25. doi: 10.1007/s12072-023-10521-0 (PMC10148013; doi:10.1007/s12072-023-10521-0)
Supplement: Supplementary file 4 — Supplementary material: List of collected data. Supplementary file4 (DOCX 122 KB) [file 12072_2023_10521_MOESM4_ESM.docx]

**Information about data collection**

Data collected from the patient records included the patient´s age and sex, date of the first symptoms, date of the first positive PCR, date of ICU admission, date of initiation of invasive mechanical ventilation, outcome, underlying liver diseases, existing comorbidities, medication prior to ICU admission (particularly angiotensin receptor blockers [ARBs] and Statins), use of corticosteroids, specific treatments such as antiviral therapy or cytokine adsorption (CytoSorb device), antibiotics, anticoagulant therapy, ketamine use, Sequential Organ Failure Assessment (SOFA) score, ventilation data (positive end-expiratory pressure [PEEP], prone positioning, PaO_2_/FiO_2_ ratio), ECMO, acute kidney failure, requirement for renal replacement therapy, thromboembolic events, onset of septic shock [MAP<65 mmHg], need for vasopressors, body mass index [BMI] and laboratory tests: liver chemistry results, interleukin-6 [IL-6], D-dimers, platelet count, prothrombin time [PT], fibrinogen, lymphocyte count, C-reactive protein [CRP], serum procalcitonin [PCT], ferritin, creatine kinase [CK], LDH, myoglobin, ABO blood type, Rh(D) phenotypes (Rh positive and negative), blood product transfusions (red blood cells [RBC] or other blood components e.g. fresh frozen plasma [FFP]), duration of mechanical ventilation and length of ICU stay (in days). In case of patients transferred from another hospital, laboratory values on initial admission to the transferring hospital were extracted from the previous patient record.

"SAVE-Berlin@COVID19" concept

**Level 1:** Highest level-of-care, ICU capable of providing care for even the most critically ill COVID-19 patients, including extracorporeal membrane oxygenation (ECMO) support.

**Level 2:** High level-of-care, ICU capable of providing COVID-19 patients critical care beds and services, including basic monitoring, differentiated catecholamine therapy, controlled invasive ventilatory support (via endotracheal tube or tracheal cannula), 24/7 invasive respiratory support for acute respiratory failure, advanced monitoring (echocardiography, trans-pulmonary thermodilution, etc.), positioning therapy (e.g., prone positioning), and organ replacement therapy (e.g., renal replacement therapy).

**Level 3:** Intensive care units capable of providing critical care beds and services for non-COVID-19 patients.
